# Supplementary material for: Physics constrained unsupervised deep learning for rapid, high resolution scanning coherent diffraction reconstruction
Source: Sci Rep. 2023 Dec 21;13:22789. doi: 10.1038/s41598-023-48351-7 (PMC10733394; doi:10.1038/s41598-023-48351-7)
Supplement: Supplementary file 1 — Supplementary Information. [file 41598_2023_48351_MOESM1_ESM.pdf]

1 Supplemental materials

1.1 Tables and figures

Supplementary Table 1: Extension of Table 2 into a full ablation study encompassing the baseline supervised model (PtychoNN, top row), PtychoPINN (bottom row), and two ablated versions of PtychoPINN, each containing one of the two defining features of the model (namely, ptychographic overlap constraints and the PINN/unsupervised structure).

		Lines		GRF		Large features	
**Feature set**	**Metric**	ϕ	A	ϕ	A	ϕ	A
{}[1]	MAE	-	0.201	0.0335	0.0153	0.219	0.0038
	PSNR (dB)	-	59.6	75.6	82.4	56.7	92.9
	FRC50 (pixel^{-1})	-	22.0	64.0	65.2	23.4	34.0
PINN	MAE	-	0.195	0.0859	0.0341	0.622	0.00581
	PSNR (dB)	-	59.7	67.5	75.4	50.3	88.8
	FRC50 (pixel^{-1})	-	22.0	29.7	64.7	8.2	13.9
overlaps	MAE	-	0.0755	0.0332	0.0158	0.187	0.00352
	PSNR (dB)	-	68.6	75.7	82.2	58.5	93.8
	FRC50 (pixel^{-1})	-	65.8	63.5	65.0	27.6	35.9
PINN,overlaps[2]	MAE	-	**0.0473**	**0.0109**	**0.00507**	**0.149**	**0.00303**
	PSNR (dB)	-	**72.6**	**85.2**	**91.9**	**60.6**	**95.0**
	FRC50 (pixel^{-1})	-	**165.4**	**171.5**	**171.3**	**93.7**	**38.7**

[1]supervised baseline

[2]full PtychoPINN

1.2 Plotting details

All amplitudes images are plotted with an auto-scaled color map. Because the model introduces a training run-specific normalization factor into reconstructed amplitudes, we omit numerical scales in the presentation of amplitude images.

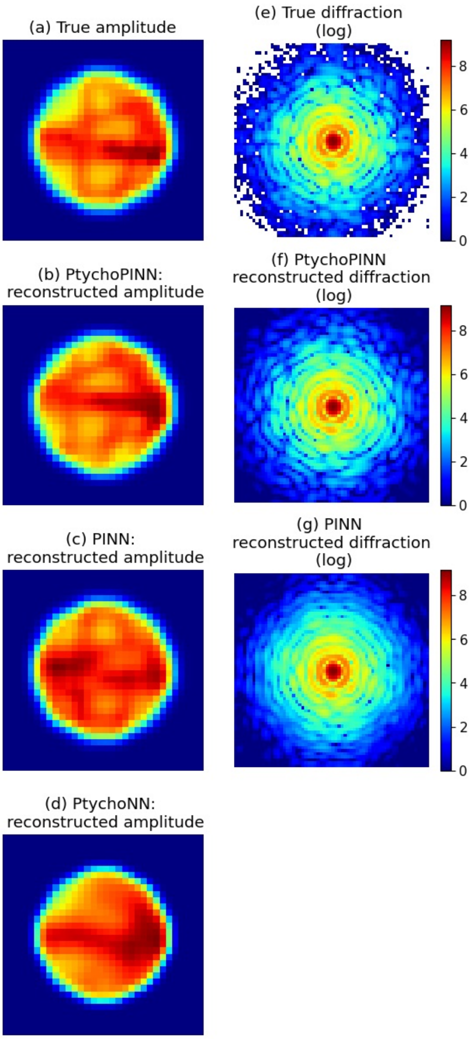

Supplementary Figure 1: *PINN and parity.* Comparison of real-space and diffraction reconstructions from PtychoPINN (b) and the basic PINN (c) with no overlap constraints. Note that both PtychoPINN and the basic PINN reconstruct small features, but only PtychoPINN resolved the inversion degeneracy correctly. The supervised-training baseline (d) produces a reconstruction that has the correct asymmetry, but is considerably blurred.
